# Multidrug Resistant CTX-M-Producing* Escherichia coli*: A Growing Threat among HIV Patients in India

**DOI:** 10.1155/2016/4152704

**Published:** 2016-03-30

**Authors:** Kesavaram Padmavathy, Krishnan Padma, Sikhamani Rajasekaran

**Affiliations:** ^1^Department of Microbiology, Research Laboratory for Oral and Systemic Health, Sree Balaji Dental College and Hospital, BIHER, Bharath University, Chennai 600100, India; ^2^Department of Microbiology, Dr. ALM PGIBMS, University of Madras, Chennai 600113, India; ^3^Government Hospital of Thoracic Medicine, Chennai 600047, India

## Abstract

Extended Spectrum *β*-Lactamases (ESBLs) confer resistance to third-generation cephalosporins and CTX-M types have emerged as the most prominent ESBLs worldwide. This study was designed to determine the prevalence of CTX-M positive ESBL-producing urinary* E. coli* isolates from HIV patients and to establish the association of multidrug resistance, phylogeny, and virulence profile with CTX-M production. A total of 57 ESBL producers identified among 76* E. coli* strains isolated from HIV patients from South India were screened for *bla*_CTX-M_, AmpC production, multidrug resistance, and nine virulence associated genes (VAGs),* fimH*,* pap*,* afa/dra*,* sfa/foc*,* iutA*,* fyuA*,* iroN*,* usp,* and* kpsMII.* The majority (70.2%) of the ESBL producers harbored *bla*_CTX-M_ and were AmpC coproducers. Among the CTX-M producers, 47.5% were found to be UPEC, 10% harbored as many as 7 VAGs, and 45% possessed* kpsMII*. Multidrug resistance (CIP^R^SXT^R^GEN^R^) was significantly more common among the CTX-M producers compared to the nonproducers (70% versus 41.2%). However, 71.4% of the multidrug resistant CTX-M producers exhibited susceptibility to nitrofurantoin thereby making it an effective alternative to cephalosporins/fluoroquinolones. The emergence of CTX-M-producing highly virulent, multidrug resistant uropathogenic* E. coli* is of significant public health concern in countries like India with a high burden of HIV/AIDS.

## 1. Introduction

Uropathogenic* Escherichia coli* (UPEC), a subset of the extraintestinal pathogenic* E. coli* (ExPEC), is the principal etiological agent of community onset urinary tract infection (UTI) accounting for substantial morbidity and medical costs worldwide. Recent studies have shown that ExPEC isolates have increasingly become resistant to the frontline antibiotics including the cephalosporins, fluoroquinolones, and trimethoprim-sulfamethoxazole [1]. Emergence of *β*-lactam resistance mediated by Extended Spectrum *β*-Lactamase (ESBLs, e.g., CTX-M types) and AmpC cephalosporinases (especially plasmid mediated AmpC enzymes, e.g., CMY types) is a major global problem [2]. ESBL mediated resistance has been increasingly reported among urinary* Escherichia coli* isolated from HIV patients in India [3, 4].

ESBL-producing isolates, especially CTX-M-producing* E. coli,* exhibit an alarming trend in coresistance to other classes of antibiotics [5–7]. Among strains of ExPEC, eleven serogroups (O1, O2, O4, O6, O7, O8, O16, O18, O25, O50, and O75) are associated with UTI and they generally belong to the virulent phylogroup B2 and to a lesser extent phylogroup D that elaborate an array of virulence factors including adhesins, protectins, toxins, and iron acquisition systems. Previous studies have reported that multidrug resistant* E. coli* strains are significantly associated with decreased virulence, non-B2 phylogenetic lineage, and host immunocompromised status [8, 9]. Also, many studies have documented a strong association between CTX-M production and decreased virulence [10–12]. Nevertheless, emergence of ESBL-producing ExPEC with a high virulence potential is of public health concern [13]. There is paucity of information on the virulence status and drug resistance profile of CTX-M-producing* E. coli* particularly infecting the HIV population in our geographical setting. Hence, this study was designed to assess the prevalence of CTX-M producers among the UPEC isolates from HIV patients and to establish the association of phylogeny, multidrug resistance, and virulence profile with CTX-M production.

## 2. Materials and Methods

### 2.1. Bacterial Isolates

A total of 76 nonrepetitive urinary* E. coli* isolated from HIV patients (with recent exposure to fluoroquinolones/3G cephalosporins, with CD4 count <350 cells/mm^3^ and not on antiretroviral therapy, with community onset UTI) from Chennai, South India, were included in the study.

### 2.2. Detection of ESBL and AmpC Production and Multidrug Resistance

ESBL production was confirmed using cefotaxime (CTX), ceftazidime (CAZ), and cefepime (FEP) alone and in combination with clavulanic acid (CLA) as per clinical and laboratory standards institute (CLSI) guidelines [14]. PCR detection of *bla*_CTX-M_ was performed among ESBL-producing urinary* E. coli* isolates from HIV patients [15]. Detection of AmpC phenotype was carried out by boronic acid method [16] and confirmed by AmpC disc test [17]. Antibiotic susceptibility testing was performed as per CLSI to assess the resistance pattern to non-beta-lactam antibiotics [14].* E. coli* ATCC 25922 was included as the control. CTX-M-producing isolates that were resistant to all the three tested antimicrobial classes (fluoroquinolones, ciprofloxacin; folate pathway inhibitor, cotrimoxazole; and aminoglycosides, gentamicin (CIP^R^SXT^R^GEN^R^)) were designated as multiple drug resistant isolates.

### 2.3. Phylogrouping and O Serogrouping

Phylogenetic grouping was done by multiplex PCR (group A (*chuA* ^−^,* yjaA* ^+/−^, and* TspE4C2* ^−^), group B1 (*chuA* ^−^, *yjaA* ^+/−^, and* TspE4C2* ^+^), group B2 (*chuA* ^+^,* yjaA* ^+^, and* TspE4C2* ^+/−^), and group D (*chuA* ^+^,* yjaA* ^−^, and* TspE4C2* ^+/−^)) [18]. O serogrouping was carried out by the traditional antiserum technique at the National* Salmonella* &* Escherichia* Centre, Central Research Institute, Kasauli, Himachal Pradesh, India.

### 2.4. Virulence Profiling

The isolates were screened for 9 virulence associated genes linked with increased clinical severity:* pap* [PapA and PapC],* sfa/foc* [S and F1C fimbriae],* afa/dra* [Dr-binding afimbrial adhesins] [19],* iutA* [aerobactin receptor],* iroN* [salmochelin receptor]*, fyuA* [aerobactin] [20],* usp* [uropathogenic specific protein] [21],* kpsMII* [group 2 capsule synthesis] [22], and* FimH* [type I fimbriae] [23] by PCR. Haemolysin production was assessed on sheep blood agar plates.

### 2.5. DNA Sequencing

DNA sequencing was performed using Applied Biosystem 3130 Genetic Analyser with ABI PRISM BigDye Terminators version 3.1 (at GenOmb Biotechnologies, Pune, India) to establish positive controls and to confirm the identity of the amplicons. The nucleotide sequences were submitted to the GenBank database under the following accession numbers: *bla*_CTX-M-15_ (HQ284192),* iroN* (HQ013325),* fyuA* (HQ013326),* iutA* (HM992940),* chuA* (HQ284193),* yjaA* (HQ284194)*, TspE4C2* (HQ284195),* papC* (HQ165752),* sfa* (HQ188690),* afa* (HQ284190),* usp* (HQ284187), and* kpsmII* (HQ284189).

### 2.6. Statistical Methods

Comparisons of proportions between the CTX-M producers and nonproducers were tested using the Chi-square test with Yates correction. Comparison of virulence scores was assessed by Mann-Whitney *U* test. *p* < 0.05 was considered statistically significant.

This study was reviewed and approved by the institutional human ethical committee, Dr. ALM PGIBMS, University of Madras, India.

## 3. Results

Of the 57 ESBL producers screened in this study, 54 (94.7%) were AmpC producers. The majority (40/57; 70.2%) of the ESBL positive isolates were found to harbor *bla*_CTX-M._ and were AmpC coproducers.

### 3.1. Phylogenetic Status and Virulence Profile

The majority of the ESBL producers belonged to the phylogroup D (27/57, 47.4%) while phylogroup B2 was the least represented (3/57, 5.3%). No significant association was observed between CTX-M^+^ and a specific phylogroup. The distribution of phylogroups among the CTX-M^+^ and CTX-M^−^ isolates was phylogroup D (20/40, 50% versus 7/17, 41.2%), A (9/40, 22.5% versus 7/17, 41.2%), B1 (9/40, 22.5% versus 2/17, 11.8%), and B2 (2/40, 5% versus 1/17, 5.9%), respectively. Of the 9 VAGs screened,* fimH* was the most predominant and* sfa/foc* was the least detected VAG among both the CTX-M producers and nonproducers (Table 1). None of the CTX-M producers were found to harbor* afa.* Compared with the CTX-M nonproducers, CTX-M-producing isolates were found to be significantly enriched for* kpsMII* (*p* = 0.048), while no statistical difference was observed in the incidence of other VAGs. Maximum virulence score of 7 was exhibited more commonly among the CTX-M producers compared to the nonproducers (10% versus 5.8%). Nonetheless, no statistical difference was observed between the mean virulence score of the CTX-M producers and nonproducers (3.95 versus 3.41, Mann-Whitney *U* test, *p* = 0.293) (Table 1).

### 3.2. UPEC

When the UPEC status of the isolates was analyzed based on the operational definition of Johnson et al. [9], that is, presence of ≥2 of the following 5 VAGs,* pap*,* sfa/foc*,* afa/dra, iutA,* and* kpsMII*, it was found that 19/40 (47.5%) CTX-M-producing isolates qualified as UPEC (mean = 2.53), while 5/17 (29.4%) of the CTX-M nonproducers were designated as UPEC (mean = 2.4). Of note, there was no statistical difference between the CTX-M producers and nonproducers with regard to the mean scores (Mann-Whitney *U* test, *p* = 0.66975). Majority of the UPEC isolates of both categories, CTX-M producers (15/19, 79%) and nonproducers (4/5, 80%), belonged to the phylogroup D.

### 3.3. Multidrug Resistance

When compared to the CTX-M nonproducers, CTX-M-producing isolates were found to be more resistant to CIP (92.5% versus 58.8%) and GEN (75% versus 47.1%). All the study isolates were found to exhibit resistance to SXT, but increased susceptibility was observed towards IPM (100%) and NIT (77.2%). Further, multidrug resistance (CIP^R^SXT^R^GEN^R^) was more common among the CTX-M producers compared to the nonproducers (70% versus 41.2%) (Table 1). It is noteworthy that 20/28 (71.4%) of these CTX-M-producing multidrug resistant (CIP^R^SXT^R^GEN^R^) isolates were susceptible to nitrofurantoin (NIT).

### 3.4. Serogroups

Among the CTX-M producers, 27.5% (11/40) of the isolates belonged to the serogroup O25, while 5.9% (1/17) of the CTX-M nonproducers belonged to the serogroup O25. Also, the majority (90.9%) of the *bla*_CTX-M^+^_ serogroup O25 isolates belonged to the virulent phylogroups (D (81.8%) and B2 (9.1%)), while 9.1% were of phylogroup A. Of the 11 *bla*_CTX-M^+^_ serogroup O25 isolates, 100% were resistant to SXT and CIP and 81.8% were resistant to GEN; none of the isolates was resistant to imipenem (IPM). Also, 9 (81.8%) of the *bla*_CTX-M^+^_ serogroup O25 isolates were found to exhibit multidrug resistance (CIP^R^SXT^R^GEN^R^), while 65.5% of the *bla*_CTX-M^+^_ serogroup non-O25 isolates were found to be multidrug resistant. Of the 9 multidrug resistant *bla*_CTX-M^+^_ serogroup O25 isolates that were analyzed, 7 (77.8%) were designated as UPEC with a mean score of 2.9.

## 4. Discussion

The EAU (European Association of Urology) recommends the use of fluoroquinolones, cephalosporins group 3a/b, for complicated UTI and urosepsis [24]. However, various reports have documented that the emergence of ESBL/AmpC-producing* E. coli* is a major public health problem and a growing challenge to patient care. Also, ESBL-producing* E. coli* is designated as a priority drug resistant microbe by the Infectious Diseases Society of America (IDSA) [25]. CTX-M-producing* E. coli* has emerged worldwide as the leading cause of community onset of UTI in the era of antibiotic resistance [26]. In line with these findings, we report that 70.2% of the ESBL-producing* E. coli* isolates recovered from HIV patients with UTI were found to harbor *bla*_CTX-M_ which is a matter of concern.

In line with the previous reports, the majority (95%) of our CTX-M producers belonged to the non-B2 phylogenetic groups, predominantly group D (50%) [10]. However, another study had reported that CTX-M-producing* E. coli* causing UTI, predominantly (57.9%), belong to the low-virulence phylogenetic group B1 [27]. A few recent reports have documented an undoubted link between CTX-M production and reduced virulence [10–12]. Also, previous studies have documented that bacterial strains acquire antibiotic resistance determinants at the expense of their virulence determinants and exhibit a multidrug resistant phenotype [28]. In contrast, our results indicate a high incidence of multidrug resistant, virulent* E. coli* of phylogroup D in our geographical area. It is noteworthy that 47.5% of the CTX-M producers were found to be UPEC, 10% had as many as 7 VAGs, and 45% harbored* kpsMII*, encoding Group II capsule synthesis. Our results suggest that these CTX-M-producing isolates had a high intrinsic virulence potential. This ascertains the fact that they evade the limited host defences and easily establish an infection in an immunocompromised host. This is in line with another study that reported that CTX-M-producing strains from community outbreaks possess an array of virulence factors that contribute to the fitness and success of these emerging multidrug resistant strains [29]. However, no clonal relationship was observed among our study isolates (data not shown).

Bingen-Bidois et al. had suggested that the concomitant presence of* papC* and* fyuA* might serve as the minimal requisite for the passage of the ExPEC from the primary focus of infection, the kidney, into the blood stream [30]. A previous study had reported that the presence of plasmid encoded aerobactin was significantly associated with antimicrobial resistance among urosepsis isolates from compromised patients [31]. We propose that ESBL-producing* E. coli* strains (both the CTX-M producers (27.5%) and nonproducers (11.8%)) carrying* pap, fyuA*, and* iutA* constitute a highly uropathogenic genotype that can have a selective advantage for causing urosepsis/bacteremia in these immunocompromised patients.

Currently, fluoroquinolones are widely used in the empirical therapy of UTI. Also, fluoroquinolones are the drug of choice for the treatment of infections caused by ESBL-producing organisms [7]. Nevertheless, a recent study has reported a strong association between ESBL production and fluoroquinolone resistance [32]. In our study, the resistance rate for CIP and GEN in CTX-M producers was remarkably higher than in nonproducers. This is in agreement with other studies that report an increased resistance towards fluoroquinolones, aminoglycosides, and folate pathway inhibitors [7, 33–35]. Though CTX-M-producing isolates showed significant reduction in susceptibility to multiple antibiotics, NIT and IPM retained significant activity.

SXT is routinely being administered to all the HIV subjects as a prophylactic drug for* Pneumocystis carinii* (*jiroveci*) pneumonia (PCP) and as a result, we observed that all our study isolates exhibited resistance to SXT. Conversely, none of the study subjects have been exposed to carbapenems which is reflected in the 100% susceptibility of the* E. coli* isolates towards IPM.

A short course of NIT has proven to be clinically and microbiologically effective in the treatment of uncomplicated cystitis in women [36]. In line with other reports, we found that majority of our ESBL producers (77.2%) were susceptible to NIT (Asha Pai et al. (88.94%) [37], Garau (71.3%) [38], Muratani & Matsumoto (>90%) [34], and Prakash et al. (73.9% of CTX-M producers) [33]). An interesting finding is that NIT was found to be effective against the multidrug resistant CTX-M producers (71.4%) suggesting that NIT may be an effective alternative to cephalosporins and fluoroquinolones.

Multidrug resistance is considered to be a common phenomenon among the CTX-M producers especially towards aminoglycosides, fluoroquinolones, and trimethoprim-sulfamethoxazole [5, 6]. In line with these reports, a high proportion (70%) of our CTX-M producers were found to be multidrug resistant (CIP^R^SXT^R^GEN^R^) suggesting the possibility of treatment failure. Previous antibiotic exposure, especially to cephalosporins and fluoroquinolones, serves as a major risk factor associated with infections caused by ESBL-producing* E. coli* [39–42]. The selection force exerted by exposure to *β*-lactams especially to CTX and CAZ fuels the emergence of divergent, multidrug resistant CTX-M clones. In our study, all the subjects were recently exposed to cephalosporins/fluoroquinolones (for the treatment of lower respiratory infection (LRI) and acute gastroenteritis (AGE), resp.) which might have possibly enhanced the emergence, persistence, and predominance of ESBL- (especially, CTX-M-) producing* E. coli* in the gastrointestinal tract. The intestine serves as the reservoir for the CTX-M producers which in turn colonize and infect the urinary tract in an ascending fashion.

Nearly 28% of the CTX-M producers belonged to the serogroup O25, of which the majority (81.8%) were of phylogroup D and one isolate (9.1%) was of phylogroup B2. Currently, highly virulent, multidrug resistant clone of* E. coli* serotype O25b:H4 belonging to multilocus sequence type 131 (ST131) phylogenetic group B2 producing CTX-M15 has been linked to community onset antimicrobial-resistant infections worldwide including the Indian subcontinent [43–46]. The VAGs commonly described in ST131* E. coli* include* iha*,* fimH*,* sat*,* kpsMII*,* fyuA*,* iutA*,* usp*,* traT*,* ompT,* and* malX.* A recent study had reported that non-ESBL-producing* E. coli* ST131 isolates were more competitive and virulent than CTX-M-producing* E. coli* ST131 isolates. However, *bla*_CTX-M_ positive transconjugants were equally competitive as their susceptible hosts which in turn would favor the global dissemination of CTX-M-producing* E. coli* ST131 isolates [47]. Though we did not screen specifically for ST131, one fluoroquinolone resistant ESBL/AmpC-producing *bla*_CTX-M^+^_ serotype O25 isolate that belonged to the phylogroup B2 exhibited multidrug resistance (Cip^R^Co^R^G^R^) and was multivirulent (*pap* ^*+*^,* fimH* ^*+*^,* fyuA* ^*+*^,* iutA* ^*+*^,* kpsMII* ^*+*^,* usp* ^*+*^,* iroN* ^*+*^,* afa* ^−^, and* sfa/foc* ^−^). However, further analysis is needed to confirm whether it belongs to the sequence type 131, a multivirulent clone that has been reported to spread across continents. Of note, this isolate was found to be susceptible to NIT.

## 5. Conclusions

Our results demonstrate a predominance of multidrug resistant CTX-M-producing* E. coli* of serotype O25, phylogroup D, with multivirulence in our geographical setting (with a high prevalence of HIV infection). We underline the need for continuous surveillance of the emergence and spread of highly virulent CTX-M-producing* E. coli* in a densely populated country such as India. Dissemination of highly virulent, multidrug resistant CTX-M-producing* E. coli* is a cause of concern and needs to be considered in the empirical management of UTI among HIV patients. Prudent use of antibiotics may serve as an important measure that would reduce antibiotic pressure which in turn could suppress the selection of these multidrug resistant strains.

## Figures and Tables

**Table 1:** VAGs/characteristics among the CTX-M producers (*n* = 40) and nonproducers (*n* = 17).

VAGs/resistance	Number (%) of strains positive for the VAG/characteristic			
	ESBL producers (*n* = 57)	CTX-M producers (*n* = 40)	Non-CTX-M producers (*n* = 17)	*p* value
*pap*	19 (33.3)	16 (40)	3 (17.7)	0.1833
*sfa/foc*	1 (1.8)	0 (0)	1 (5.9)	0.6564
*afa/dra*	3 (5.3)	2 (5)	1 (5.9)	0.8914
*iutA*	32 (56.1)	24 (60)	8 (47.1)	0.5425
*fyuA*	45 (78.9)	32 (80)	13 (76.5)	0.7649
*iroN*	20 (35.1)	15 (37.5)	5 (29.4)	0.7779
*kpsMII*	21 (36.8)	18 (45)	3 (17.7)	0.0486^a^
*usp*	19 (33.3)	11 (27.5)	8 (47.1)	0.2602
Haemolysis	3 (5.3)	2 (5)	1 (5.9)	0.8914
*AmpC*	54 (94.7)	40 (100)	14 (82.4)	0.0374^b^
CIP^R^	47 (82.5)	37 (92.5)	10 (58.8)	0.0037^c^
SXT^R^	57 (100)	40 (100)	17 (100)	1
GEN^R^	38 (66.7)	30 (75)	8 (47.1)	0.0818
CIP^R^SXT^R^GEN^R^	35 (61.4)	28 (70)	7 (41.2)	0.0403^d^

VAGs: virulence associated genes, CIP^R^: resistant to CIP, SXT^R^: resistant to SXT, GEN^R^: resistant to GEN, and CIP^R^SXT^R^GEN^R^: multidrug resistance.

^a^ *p* < 0.05, odds ratio: 3.8182, and 95% CI: 0.9473 to 15.389.

^b^ *p* < 0.05, odds ratio: 32.3665, and 95% CI: 2.607 to 401.8328.

^c^ *p* < 0.05, odds ratio: 8.6333, and 95% CI: 1.8839 to 39.5634.

^d^ *p* < 0.05, odds ratio: 3.3333, and 95% CI: 1.0252 to 10.8382.
